# TmToll-7 Plays a Crucial Role in Innate Immune Responses Against Gram-Negative Bacteria by Regulating 5 AMP Genes in *Tenebrio molitor*

**DOI:** 10.3389/fimmu.2019.00310

**Published:** 2019-03-12

**Authors:** Soyi Park, Yong Hun Jo, Ki Beom Park, Hye Jin Ko, Chang Eun Kim, Young Min Bae, Bobae Kim, Sung Ah Jun, In Seok Bang, Yong Seok Lee, Yu Jung Kim, Yeon Soo Han

**Affiliations:** ^1^Division of Plant Biotechnology, College of Agriculture and Life Sciences, Institute of Environmentally-Friendly Agriculture Chonnam National University, Gwangju, South Korea; ^2^Institute of Medical Science, University of Toronto, Toronto, ON, Canada; ^3^Department of Biological Science, Hoseo University, Asan, South Korea; ^4^Department of Life Science and Biotechnology, College of Natural Sciences, Soonchunhyang University, Asan, South Korea; ^5^Department of Chemistry and Biochemistry, College of Natural Sciences, California State University, San Bernardino, CA, United States

**Keywords:** Toll-7, *Tenebrio molitor*, microbial infection, RNAi, antimicrobial peptides, AMP assay

## Abstract

Although it is known that the *Drosophila* Toll-7 receptor plays a critical role in antiviral autophagy, its function in other insects has not yet been reported. Here, we have identified a Toll-like receptor 7 gene, *TmToll-7*, in the coleopteran insect *T. molitor* and examined its potential role in antibacterial and antifungal immunity. We showed that *TmToll-7* expression was significantly induced in larvae 6 h after infection with *Escherichia coli* and *Staphylococcus aureus* and 9 h after infection with *Candida albicans*. However, even though *TmToll-7* was induced by all three pathogens, we found that *TmToll-7* knockdown significantly reduced larval survival to *E. coli*, but not to *S. aureus*, and *C. albicans* infections. To understand the reasons for this difference, we examined the effects of *TmToll-7* knockdown on antimicrobial peptide (AMP) gene expression and found a significant reduction of *E. coli*-induced expression of AMP genes such as *TmTenecin-1, TmDefensin-1, TmDefensin-2, TmColeoptericin-1*, and *TmAttacin-2*. Furthermore, *TmToll-7* knockdown larvae infected with *E. coli* showed significantly higher bacterial growth in the hemolymph compared to control larvae treated with *Vermilion* dsRNA. Taken together, our results suggest that TmToll-7 plays an important role in regulating the immune response of *T. molitor* to *E. coli*.

## Introduction

Toll and Toll-like receptors in *Drosophila* and mammals play a major role in innate immunity (1). So far, in *Drosophila*, nine Toll receptors (Toll, 18-Wheeler, and Toll-3 to Toll-9) have been identified (2). Toll, which was the first of these receptors to be discovered, was initially noted for its role in dorsal-ventral patterning in *Drosophila* embryos (3) and was later shown to participate in the immune response to fungi and Gram-positive bacteria in larvae and adults (4). In addition to its role in salivary gland morphogenesis (5), the 18-wheeler or Toll-2 receptor is required for antibacterial defense in larvae but dispensable for immune responses in adults (6). Among what is known so far of the remaining Toll receptors is that Toll-7 and Toll-8 may have developmental functions that are redundant with 18-wheeler (7), and although they do not induce antibacterial responses in flies, Toll-7 is involved in antiviral defense (8) and Toll-8 acts as a negative regulator of antibacterial responses in respiratory epithelial cells (9). Furthermore, in addition to its role in development and immunity, Toll-7 along with Toll-6 have been shown to have neurotrophic functions and are required for locomotion behavior, motor axon targeting, and neuronal survival (10). More recently, Toll-6 and Toll-7 were also discovered to mediate axon and dendrite targeting during *Drosophila* olfactory circuit assembly (11). Toll-5 has been shown to activate Drosomycin and Metchnikowin expression in transfected cells (12). However, whether Toll-5 has the same function *in vivo* still remains to be determined. Finally, overexpression of Toll-9 in S2 cells and larval fat body tissues results in constitutive expression of antimicrobial peptides (13, 14). Based on these findings, it was proposed that Toll-9 may have a constitutive function in activating antimicrobial defense (13, 14). However, a later study showed that Toll-9 loss-of function-mutants could still induce AMP expression under unchallenged conditions, suggesting that Toll-9 is not required to maintain a basal antimicrobial response as initially hypothesized, but may function redundantly with other Toll proteins (15).

Overall, the structure of insect Tolls are similar to mammalian TLRs in that they have an extracellular leucine-rich repeat (LRR) domain, a transmembrane domain, and a cytoplasmic Toll/Interleukin-1 receptor (TIR) domain (16). Despite this similarity, a number of structural and functional differences exist. One notable difference is that the *Drosophila* Tolls contain cysteine-rich clusters at either, or both, the N- and C-terminal ends of the LRRs, whereas mammalian TLRs contain a single cysteine-rich cluster at the C-terminal end of the LRRs, adjacent to the plasma membrane (16–18). The one exception is Toll-9, which has a single cysteine cluster, and because its TIR domain is more similar to the mammalian TLRs than to the other eight *Drosophila* Tolls, it falls within the same phylogenetic subfamily as the TLRs rather than with other insect Tolls (16, 17, 19). Thus, it might be expected that it would share functional similarities with mammalian TLRs, which serve to initiate inflammatory pathways following recognition of conserved pathogen associated molecular patterns (PAMPs) on various microbes, including bacteria, viruses, fungi, and protozoa (2, 19–21). However, the significance of Toll-9 and its role in immunity is unclear (13, 14).

Much of what is known about Toll activation in insect immunity comes from studies with *Drosophila* Toll-1, which unlike mammalian TLRs, does not respond directly to microbial molecules, but is activated by the cytokine ligand Spätzle (Spz) (22). However, for this binding to occur, Spz must be cleaved by the serine protease Spätzle-processing enzyme (SPE), which in turn is activated by three protease cascades. Two of them, which converge at the level of the ModSP-Grass proteases, are initiated when pattern recognition receptors detect cell wall components from Gram-positive bacteria (Lysine-type peptidoglycans) and fungi (β-1,3-glucan), respectively (23). Furthermore, four other serine proteases (Spirit, Spheroide, and Sphinx1/2) are thought to function in this pathway between Grass and SPE (24). The third cascade is triggered when the protease Persephone (Psh) is activated by microbial proteases or endogenous danger signals. With the former, it is believed that Persephone can be targeted for direct cleavage by subtilisin-like serine proteases produced by Gram-positive bacteria or fungi (25, 26). With the latter, although it is unclear what signals are actually sensed by Persephone and how, one possibility is that it is able to detect abnormal proteolytic activities in the hemolymph (26). This was suggested based on the observation that Grass overexpression in transgenic flies results in Persephone-dependent Toll activation, similar to what occurs when pathogen-derived proteases are injected into flies (26). In short, all three modes of detection lead to downstream activation of SPE. However, currently it is unknown what protease directly cleaves and activates SPE in *Drosophila*, although an SPE-activating enzyme (*Tm*SAE) has been found in *T. molitor* (27, 28).

The mechanisms leading to Toll receptor activation in *T. molitor* and *Drosophila* are similar, but they also have many important differences. In both of these insects, Toll signaling is initiated when the peptidoglycan-recognition protein-SA (PGRP-SA) and Gram-negative binding protein 1 (GNBP1) recognize lysine-type peptidoglycan from Gram-positive bacteria, and GNBP3 recognizes β-1,3 from fungi (29, 30). However, in the case of the *Tenebrio* PGRP-SA/GNBP1 complex, it also recognizes *meso*-diaminopimelic acid (DAP)-type peptidoglycan, which is found in most Gram-negative bacteria and certain Gram-positive bacteria (e.g., *Bacillus* species and *L. monocytogenes*) (29). This is unlike *Drosophila*, where DAP-type peptidoglycan recognition depends on two other pattern recognition receptors, PGRP-LC and PGRP-LE, both of which are known to activate the Imd pathway (31). A further difference is that while both systems have a modular serine protease (ModSP) that functions directly downstream of PGRP-SA/GNBP1 and GNBP3, the *Tenebrio* ModSP proceeds through sequential activation of clip-domain-containing serine proteases, *Tm*-SAE and *Tm*-SPE, whereas in *Drosophila*, it activates the protease Grass, which in turn leads to activation of SPE (27, 32). In recent years, the mechanism for Spätzle activation in *T. molitor* in response to components of Gram-positive and Gram-negative bacteria and fungi has largely been determined using purified proteases (28). These studies have provided insights into the extracellular recognition mechanisms involved in Toll signaling (27, 29, 33); however, the Toll receptors themselves and their downstream components have not been characterized so far. Here, we have cloned a *Toll-like receptor 7* gene from *T. molitor* (*TmToll-7*) and examined its role *in vivo* by RNAi. Our current study reveals that TmToll-7 is important for immune mediating responses to Gram-negative bacteria *E. coli*.

## Materials and Methods

### Insect Rearing and Microbial Infection

*T. molitor* larvae were reared on wheat bran diet at 27 ± 1°C, 60 ± 5% relative humidity, and under dark conditions. All experiments were conducted with 10–12th instar larvae. To investigate the immunological function of TmToll-7 against infections, three microorganisms, including *Escherichia coli* K12, *Staphylococcus aureus* RN4220, and *Candida albicans* were used. Overnight cultures of *E. coli, S. aureus*, and *C. albicans* were grown in Luria-Bertani (LB) broth and Sabouraud Dextrose broth at 37°C, respectively. The microorganisms were harvested, washed, and suspended in phosphate-buffered saline (PBS, pH 7.0) by centrifugation at 3,500 rpm for 10 min, and the concentrations were measured at OD_600_. Finally, 10^6^ cells/μl of *E. coli* and *S. aureus* and 5 × 10^4^ cells/μl of *C. albicans* were used in infection experiments.

### Identification, Full-Length cDNA Cloning, and *in silico* Analysis of *TmToll-7*

The amino acid sequence (EEZ99327.1) of Toll-like receptor 7 (TcToll-7) in *Tribolium castaneum* was used to identify the *TmToll-7* gene from a nucleotide sequence database constructed with *T. molitor* EST and RNAseq by local-tblastn analysis (34). The *TmToll-7* nucleotide sequence was annotated with the Genbank nr database by the blastx program.

To obtain the full-length cDNA sequence of *TmToll-7* ORF cloning and 5′- and 3′-RACE, PCR was conducted with the gene specific primers listed in Table 1. All primers were designed by using the Primer 3 plus program (http://primer3plus.com/cgi-bin/dev/primer3plus.cgi). *TmToll-7* was amplified by reverse transcription-PCR (RT-PCR) using Ex Taq polymerase (TaKaRa, Japan) under the following conditions: pre-denaturation at 98°C for 3 min, denaturation at 98°C for 10 s, annealing at 53°C for 30 s, and extension at 72°C for 1 min for 35 cycles. To obtain 5′- and 3′- end sequences of *TmToll-7*, 5′- and 3′-RACE PCR was carried with the SMARTer RACE cDNA amplification kit (Clontech Laboratories, Palo Alto, CA, USA) according to the manufacturer's instructions. Conditions for *TmToll-7* RACE PCR were as follows: pre-denaturation at 94°C for 5 min, denaturation at 94°C for 30 s, annealing at 55°C for 30 s, and extension at 72°C for 30 s for 35 cycles. The RT-PCR products and nested PCR products were purified with AccuPrep ® PCR Purification Kit (Bioneer, Korea), cloned into TOPO TA cloning vector (Invitrogen Co., Carlsbad, CA), and subsequently transformed into *E. coli* DH5α competent cells and sequenced. The obtained sequences were assembled by the Cap3.dat program.

**Table 1:** Primers used in the present study.

**Name**	**Primer sequences**
TmToll-7-Set#1_Fw	5′-CGGTGAAATGGTGAGACTGA-3′
TmToll-77-Set#1_Rv	5′-TTTGAGCGCCGAACAATTAC-3′
TmToll-7-Set#2_Fw	5′-ATCGGCCCATTGTTATTCAA-3′
TmToll-7-Set#2_Rv	5′-ATTGGACAGGTGCTCGAACT-3′
TmToll-7-Set#3_Fw	5′-AACAGCACCTTCAACGGTTT-3′
TmToll-7-Set#3_Rv	5′-CAGTTAAGTTATCACACGAGATAGGC-3′
TmToll-7-5′RACE_GSP1	5′-ACGTCACGGAGAACTTCGTC-3′
TmToll-7-5′RACE_GSP2	5′-CAGCACCTGGAGCTCTTTGA-3′
TmToll-7-3′RACE_GSP1	5′-CCCACGTCATGATGCAGACT-3′
TmToll-7-3′RACE_GSP2	5′-CCCCGAGAAGATCAAGACCC-3′
TmToll-7_T7_Fw	5′-TAATACGACTCACTATAGGGT GCAACGGTTCGTTCAAAAAT-3′
TmToll-7_T7_Rv	5′-TAATACGACTCACTATAGGGT TGATGAACAAGAGCTCCACG-3′
TmToll-7_qPCR_Fw	5′-ACATGCGAGTGTTGTTCGTG-3′
TmToll-7_qPCR_Rv	5′-ACGTGTGCAAACCGTTGAAG-3′
TmTene-1_Fw	5′-CAGCTGAAGAAATCGAACAAGG-3′
TmTene-1_Rv	5′-CAGACCCTCTTTCCGTTACAGT-3′
TmTene-2_Fw	5′-CAGCAAAACGGAGGATGGTC-3′
TmTene-2_Rv	5′-CGTTGAAATCGTGATCTTGTCC-3′
TmTene-3_Fw	5′-GATTTGCTTGATTCTGGTGGTC-3′
TmTene-3_Rv	5′-CTGATGGCCTCCTAAATGTCC-3′
TmTene-4_Fw	5′-GGACATTGAAGATCCAGGAAAG-3′
TmTene-4_Rv	5′-CGGTGTTCCTTATGTAGAGCTG-3′
TmDef-1_Fw	5′-AAATCGAACAAGGCCAACAC-3′
TmDef-1_Fw	5′-GCAAATGCAGACCCTCTTTC-3′
TmDef-2_Fw	5′-GGGATGCCTCATGAAGATGTAG-3′
TmDef-2_Fw	5′-CCAATGCAAACACATTCGTC-3′
TmCole-1_Fw	5′-GGACAGAATGGTGGATGGTC-3′
TmCole-1_Rv	5′-CTCCAACATTCCAGGTAGGC-3′
TmCole-2_Fw	5′-GGACGGTTCTGATCTTCTTGAT-3′
TmCole-2_Rv	5′-CAGCTGTTTGTTTGTTCTCGTC-3′
TmAtt-1a_Fw	5′-GAAACGAAATGGAAGGTGGA-3′
TmAtt-1a_Rv	5′-TGCTTCGGCAGACAATACAG-3′
TmAtt-1b_Fw	5′-GAGCTGTGAATGCAGGACAA-3′
TmAtt-1b_Rv	5′-CCCTCTGATGAAACCTCCAA-3′
TmAtt-2_Fw	5′-AACTGGGATATTCGCACGTC-3′
TmAtt-2_Rv	5′-CCCTCCGAAATGTCTGTTGT-3′
TmCec-2_Fw	5′-TACTAGCAGCGCCAAAACCT-3′
TmCec-2_Rv	5′-CTGGAACATTAGGCGGAGAA-3′
TmTLP-1_Fw	5′-CTCAAAGGACACGCAGGACT-3′
TmTLP-1_Rv	5′-ACTTTGAGCTTCTCGGGACA-3′
TmTLP-2_Fw	5′-CCGTCTGGCTAGGAGTTCTG-3′
TmTLP-2_Rv	5′-ACTCCTCCAGCTCCGTTACA-3′
TmL27a_qPCR_Fw	5′-TCATCCTGAAGGCAAAGCTCCAGT-3′
TmL27a_qPCR_Rv	5′-AGGTTGGTTAGGCAGGCACCTTTA-3′

*Underline indicates T7 promotor sequences*.

Specific domains of TmToll-7 were analyzed with the SignalP 4.1 program (http://www.cbs.dtu.dk/services/SignalP/), InterProScan 5 program (35–37), and TMHMM 2.0 program (http://www.cbs.dtu.dk/services/TMHMM/). Phylogenetic analysis and percentage identity/distance analysis were conducted by using the Clustal X2 (38) and MEGA 6 programs (39), respectively. Human TLR4 amino acid sequences were used as an outgroup.

### Expression and Induction Patterns of *TmToll-7*

Developmental and tissue specific expression patterns of *TmToll-7* were investigated by relative quantitative PCR (qRT-PCR) method using the Exicycler Real-Time PCR Quantification System (Bioneer Co., Daejon, South Korea). To investigate developmental and tissue specific expression patterns of *TmToll-7*, samples were collected from various developmental stages including late instar larval, pre-pupal, 1–7 day old pupal, and 1–2 day old adult stages and tissues dissected from late instar larvae (integument, gut, fat body, Malpighian tubules, and hemocytes) and 5 day old adult (integument, gut, fat body, Malpighian tubules, hemocytes, ovary, and testis) individuals. To examine induction patterns of *TmToll-7* challenged by microorganisms, *E. coli* (10^6^ cells/μl), *S. aureus* (10^6^ cells/μl), and/or *C. albicans* (5 × 10^4^ cells/μl) were injected into *T. molitor* larvae and samples were collected at different time points, including 3, 6, 9, and 12 h post-injection of microorganisms. PBS-injected *T. molitor* larvae were used as a negative control.

Total RNAs were extracted by using FavorPrep™ Tri-RNA Reagent (Favorgen biotech corp., Ping-Tung, Taiwan), following which 2 μg of total RNAs were used to synthesize cDNA using AccuPower® RT PreMix (Bioneer, Korea) with Oligo (dT)_12−18_ primer on a MyGenie 96 thermal block (Bioneer, Korea). To investigate the expression levels of *TmToll-7* transcripts, relative quantitative PCR was examined by using AccuPower® 2X GreenStar qPCR Master Mix (Bioneer) with synthesized cDNAs and specific primers, TmToll-7_qPCR_Fw and TmT7_qPCR_Rv (Table 1) at an initial denaturation of 95°C for 20 s, followed by 45 cycles at 95°C for 5 s, and 60°C for 20 s. *T. molitor* ribosomal protein (*TmL27a*) was used as an internal control, and the results were analyzed by using ΔΔCt methods.

### RNA Interference Analysis

The PCR product (510 bp sequence) containing the T7 promotor sequences was amplified by Ex Taq polymerase with TmToll-7_T7_Fw and Rv primers (Table 1) using the same PCR condition mentioned above. dsRNA for *TmToll-7* was synthesized by using AmpliScribe T7-Flash Transcription Kit (Epicentre, Madison, Wisconsin, USA) and was purified by PCI (Phenol: Chloroform: Isopropyl alcohol mixture), ammonium acetate purification and ethanol precipitation. 2 μg of synthesized ds*TmToll-7* was injected into 10–11th instar larvae for gene silencing and the ds*TmVer* was used as a control.

### Effect of *TmToll-7* Knockdown in Response to Microorganisms

To investigate the effect of *TmToll-7* knockdown in response to microorganisms, 10^6^ cells/μl of *E. coli* and *S. aureus* and 5 × 10^4^ cells/μl of *C. albicans* were injected into ds*TmToll-7*-treated *T. molitor* larvae, respectively. The dead larvae were counted up to 10 days post-injection of microorganisms. Ten insects per group were used for this assay and the experiments were replicated three times.

### Effect of *TmToll-7* RNAi on AMP Expression in Response to Microorganisms

To characterize the function of *TmToll-7* on humoral innate immune response, *TmToll-7* RNAi technique was applied and microorganisms, including *E. coli, S. aureus* and *C. albicans*, were injected subsequently. Samples were collected at 24 h post-injection of microorganisms. PBS was used as an injection control and ds*TmVer*-treated *T. molitor* was used as a negative control. Temporal expression patterns of 14 antimicrobial peptide (AMP) genes, including *TmTene-1* (**Figure 6A**: *TmTenecin-1*), *TmTene-2* (**Figure 6B**: *TmTenecin-2*), *TmTene-3* (**Figure 6C**: *TmTenecin-3*), *TmTene-4* (**Figure 6D**: *TmTenecin-4*), *TmDef-1* (**Figure 6E**: *TmDefensin-1*), *TmDef-2* (**Figure 6F**: *TmDefensin-2*), *TmCole-1* (**Figure 6G**: *TmColeoptericin-1*), *TmCole-2* (**Figure 6H**: *TmColeoptericin-2*), *TmAtt-1a* (**Figure 6I**: *TmAttacin-1a*), *TmAtt-1b* (**Figure 6J**: *TmAttacin-1b*), *TmAtt-2* (**Figure 6K**: *TmAttacin-2*), *TmCec-2* (**Figure 6L**: *TmCecropin-2*), *TmTLP-1* (**Figure 6M**: *TmThaumatin-like protein-1*), and *TmTLP-2* (**Figure 6N**: *TmThaumatin-like protein-2*) were investigated by relative quantitative-PCR with AMP gene specific primers detailed in Table 1.

### *TmToll-7* RNAi Has Critical Role in Antimicrobial Activity Against *E. coli*

The effect of *TmToll-7* RNAi on antimicrobial activity against *E. coli* was investigated by using a colony formation assay (40). *E. coli* (10^6^ cells) was injected into *TmToll-7* dsRNA-treated *T. molitor* larvae and hemolymph was isolated in 100 μl 1X PBS at 24 h after *E. coli* injection. PBS-injected *T. molitor* hemolymph samples were used as an uninfected control, and dsTmVer-treated hemolymph was used as a dsRNA control. Hemolymph samples were centrifuged at 15,000 rpm at 4°C for 5 min and then the supernatants were boiled at 100°C for 5 min and centrifuged again at 15,000 rpm at 4°C for 5 min. Fifty nanogram of hemolymph samples were assayed with 10^6^ cells of *E. coli* in 1X PBS at 37°C for 2 h. 2,000-fold diluted samples on LB-agar plates were incubated at 37°C for 16 h. The colony numbers of assayed plates were then counted.

### Statistical Analysis

All experiments were performed in triplicate and all data are shown as means ± S.E. The one-way analysis of variance (ANOVA) and Tukey's multiple range tests were used to evaluate the difference between groups (*p* < 0.05).

## Results

### Full-Length cDNA Cloning and Sequence Analysis of *TmToll-7*

**Figure 1:**
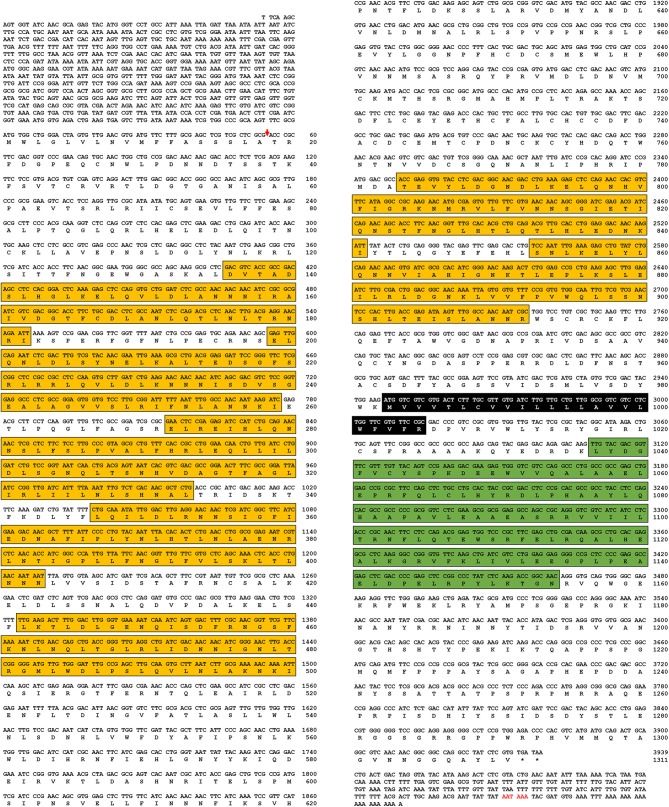
Nucleotide and deduced amino acid sequences of *TmToll-7*. Amino acid sequences of TmToll-7 were deduced by using the BLAST program and “DNA to protein translation program” provided from biophp (http://biophp.org/). Domains were analyzed by using the SignalP 4.1 program (http://www.cbs.dtu.dk/services/SignalP/), InterProScan program (http://www.ebi.ac.uk/Tools/pfa/iprscan5/), and TMHMM Server v. 2.0 program (http://www.cbs.dtu.dk/services/TMHMM-2.0/). The arrow, yellow box, black box, and green box indicate the signal peptide region (one), leucine rich repeat region (seven), transmembrane domain (one), and Toll/interleukin-1 receptor homology (TIR) domain (one), respectively.

In this study, a Toll-7 homolog from *Tenebrio molitor* (*TmToll-7*, Accession number: MK234903) was identified by an EST and RNA-seq search using the *T. castaneum* protein sequence as a query. Based on this sequence information, we cloned the corresponding full-length cDNA from *Tenebrio* larval RNA by RT-PCR and 5′- and 3′-RACE. The 6,097-bp cDNA contains an 847-bp 5′-untranslated region (UTR), a 231-bp 3′-UTR (excluding the poly-A tail), and a 3,939-bp open reading frame (ORF) encoding a protein of 1,311 amino acids (Figure 1). Domain analysis of the deduced amino acid sequence shows that TmToll-7 has an N-terminal signal peptide from amino acids 1 to 18, seven leucine-rich repeats between amino acids 135 and 889, a transmembrane domain from amino acids 980 to 1,004, and a C-terminal Toll/interleukin-1 receptor homology (TIR) domain at positions 1,036 to 1,174 (Figure 1).

**Figure 2:**
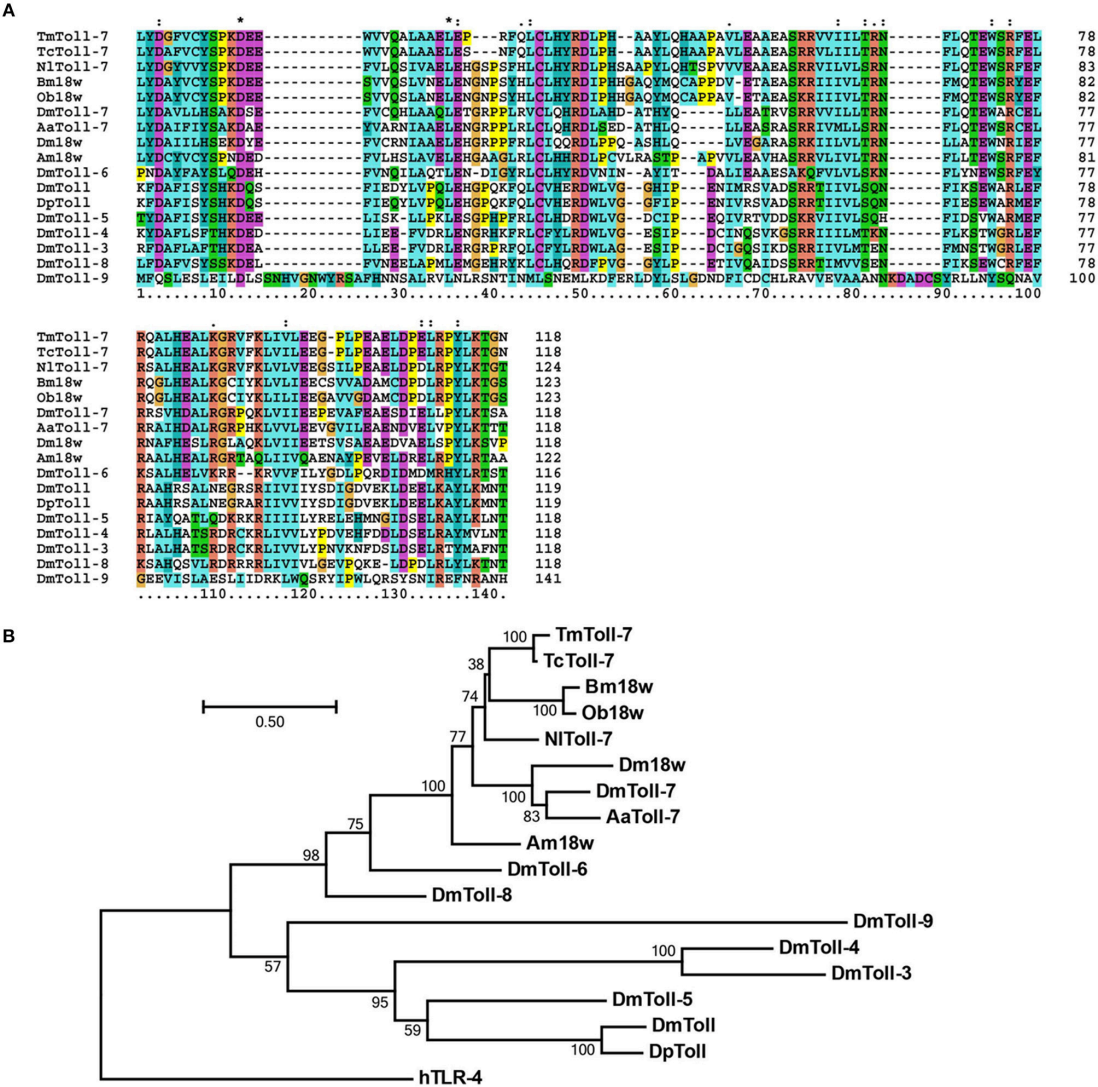
Multiple alignment and molecular phylogenetic analysis of insect TLR7s. **(A)** The highly conserved TIR domain was aligned by using ClustalX 2.1 software and the result was visualized by Gendoc software. **(B)** Molecular phylogenetic analysis was performed with whole amino acid sequences of insect TLR7s. ClustalX 2.1 software was used to perform multiple amino acid alignments before phylogenetic analysis. The phylogenic tree was constructed by MEGA 6.06 software. Bootstrap analysis of 1,000 replications are shown. *TmToll-7, T. molitor* Toll-like receptor 7 (MK234903); *TcToll-7, T. castaneum* Toll-like-7 protein (EEZ99327.1); *NlTollR-7, Nilaparvata lugens* Toll-like receptor 7 (AGK40936.1); *Bm18w, Bombyx mori* 18 wheeler (BAB85498.1); *DmToll-7, Drosophila melanogaster* Toll-7 (AAF57514.1); *Am18w, Apis mellifera* 18-wheeler precursor (NP_001013379.1); *Ob18w, Operophtera brumata* 18 wheeler (KOB69977.1); *AaToll-7, Aedes aegypti* Toll-like receptor 7 (EAT46215.1); *DmToll, Drosophila melanogaster* Toll protein (AAA28941.1); *DpToll, Drosophila pseudoobscura* Toll protein (L25390); *DmToll-3, Drosophila melanogaster* Toll-3 (AAF54021.3); *DmToll-4, Drosophila melanogaster* Toll-4 (AAF52747.3); *DmToll-5, Drosophila melanogaster* Toll-5 (AAF86227.1); *DmToll-6, Drosophila melanogaster* Toll-6 (AAF86226.1); *DmToll-8, Drosophila melanogaster* Toll-8 (AAF86224.1); *DmToll-9, Drosophila melanogaster* Toll-9 (NP_649214.1); and *HsTLR4, Homo sapiens* toll-like receptor 4 precursor (AAY82270.1).

Multiple alignment of Toll-7 homologs indicates that the TIR domain is highly conserved in insects (Figure 2A). Further, phylogenetic analysis based on the full-length amino acid sequences of TmToll-7 and other insect Toll receptors (which include *D. melanogaster* Toll-1 through Toll-9) indicates that it clusters together with the Toll-7 proteins from *T. castaneum, Nilaparvata lugens, D. melanogaster*, and *Aedes aegypti*, and with the 18W proteins from *Bombyx mori, Operophtera brumata, Apis mellifera*, and *Drosophila melanogaster* (Figure 2B). Furthermore, within this branch, TmToll-7 appears to be most closely related to TcToll-7 (93% aa identity) from *T. Castaneum*, which belongs to the same insect order as *T. molitor* (Coleoptera), with AmToll-7 (68% aa identity) from *Apis mellifera* (Hymenoptera) and Bm18W (61% aa identity) from *Bombyx mori* (Lepidoptera) being the next two closely related sequences, followed by Toll-7 (55% aa identity) and 18W (53% aa identity) from *D. melanogaster* (Diptera).

### Expression Analysis of *TmToll-7* Transcripts

**Figure 3:**
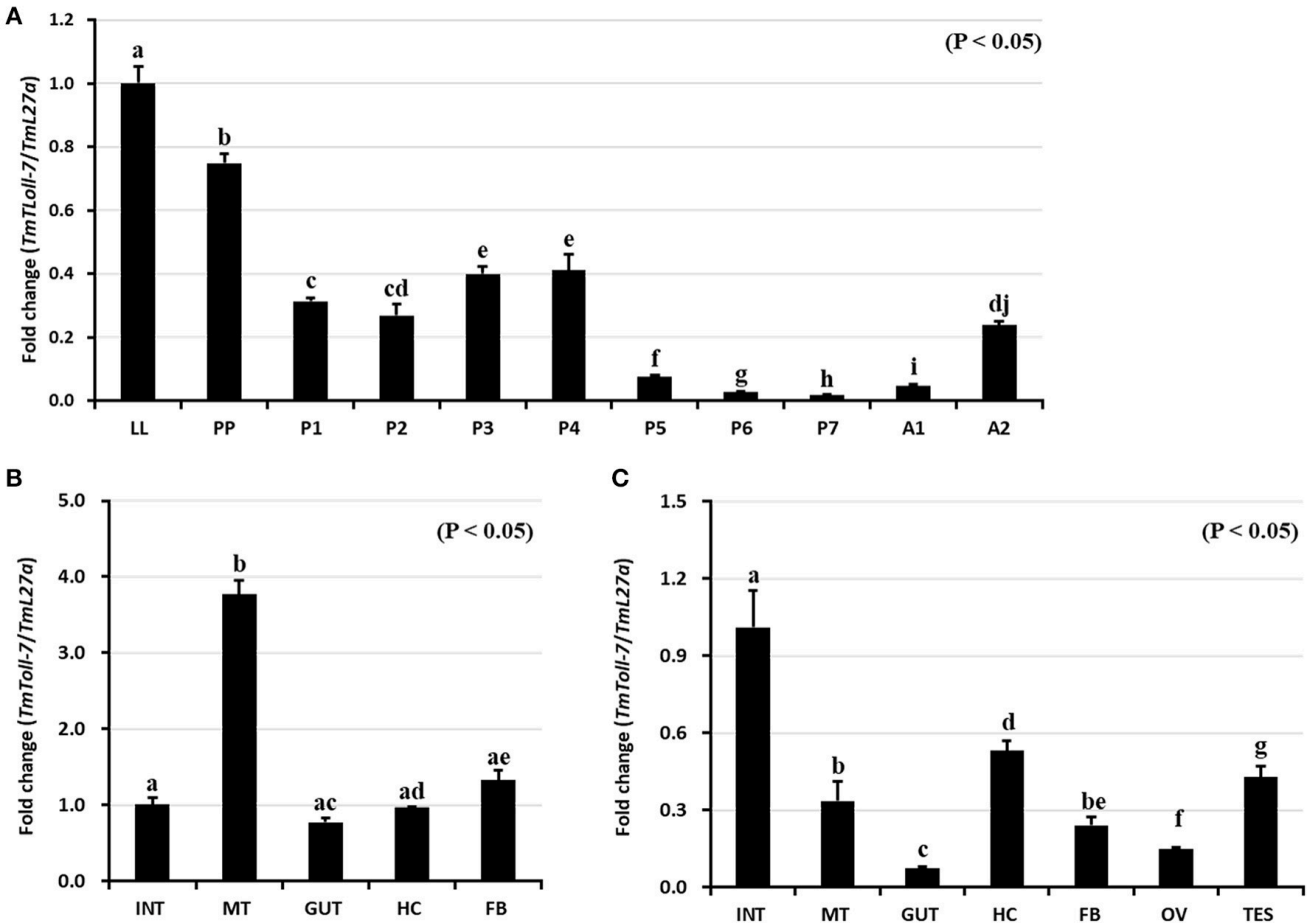
Developmental and tissue specific expression patterns of *TmToll-7*. **(A)** For developmental expression analysis of *TmToll-7*, total RNAs were isolated from late instar larvae (LL), pre-pupae (PP), 1~7 day old pupae (P1~P7), and 1~2 day old adults (A1~A2). *T. molitor* 60S ribosomal protein 27a (TmL27a) primers were used as an internal control. *TmToll-7* was highly expressed in late-instar larval and prepupal stage (*N* = 3). Tissue specific expression patterns of *TmToll-7* in late instar larvae **(B)**, and adults **(C)**. Total RNAs were isolated from various tissues, including integument (INT), Malpighian tubule (MT), gut (GUT), hemocytes (HC), and fat body (FB) in late instar larvae and integument (INT), Malpighian tubule (MT), gut (GUT), hemocytes (HC), fat body (FB), ovary (OV) and testis (TES) from 5th day old adults. Signals were detected by using relative quantitative PCR and ΔΔCT method was used to analyze tissue specificity (*N* = 3).

We investigated expression pattern of *TmToll-7* by qRT-PCR at four different developmental stages (late-instar larvae, pre-pupae, 1 to 7-day-old pupae, and 1 to 5-day-old adults). Our results showed that *TmToll-7* expression level was high in the late-instar larval stage, but then gradually decreased between the pre-pupal and adult stages (Figure 3A). We further examined *TmToll-7* transcript levels in different tissues of late-instar larvae and adults. As shown in Figure 3B, *TmToll-7* expression was relatively high in the Malpighian tubules of late-instar larvae and low in the fat body, integument, hemocytes, and gut. In comparison, in adult tissues, *TmToll-7* was predominantly expressed in the integument (albeit at low levels similar to those found in late-instar larvae) and to a lesser extent in other tissues, including testes and hemocytes (Figure 3C).

**Figure 4:**
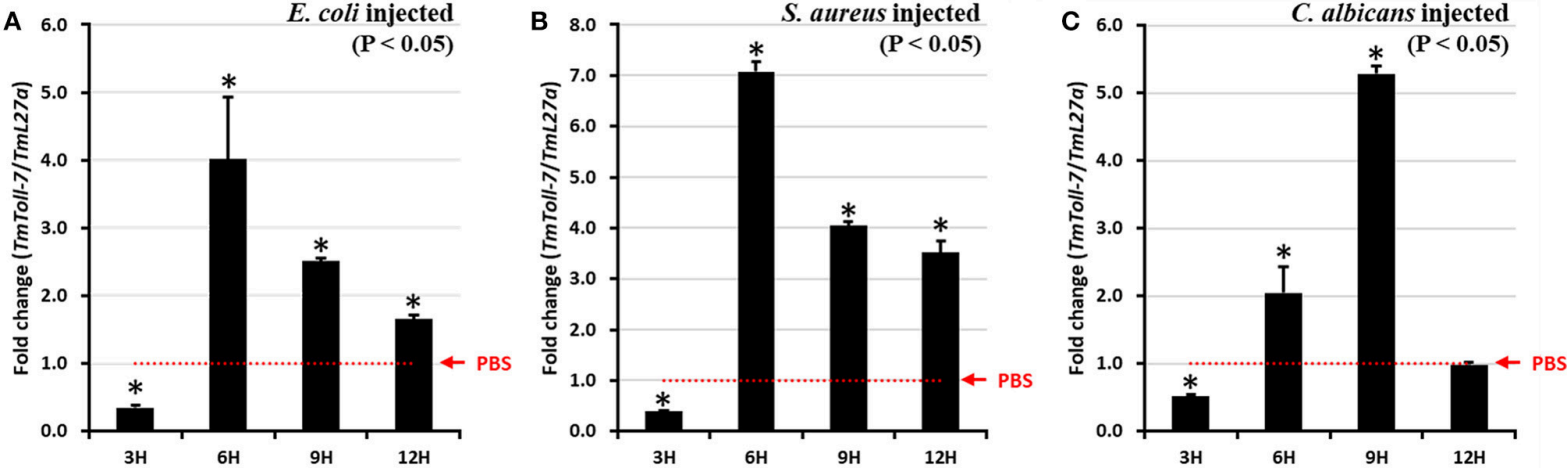
Temporal expression patterns of *TmToll-7* in late instar larvae whole body. *E.coli* **(A)**, *S. aureus* **(B)**, and *C. albicans* **(C)** were injected into 10–11th instar larvae and total RNAs were isolated from whole-body at 3, 6, 9, and 12 h post injection of microorganisms. PBS was used as an injection control. The results show that *TmToll-7* was highly induced at 6 h post injection of *E. coli* and *S. aureus*, and 9 h after injection of *C. albicans* (*n* = 3).

Next, to determine whether *TmToll-7* expression is regulated in response to immune challenge, we further examined temporal changes in *TmToll-7* mRNA expression in *T. molitor* larvae after infection with either Gram-negative (*E. coli*) or Gram-positive (*S. aureus*) bacteria, or fungus (*C. albicans*). Briefly, this was done by isolating total RNA from control and immune-challenged larvae (tenth or eleventh instar) at 3, 6, 9, and 12 h post-infection, followed by reverse-transcription and qRT-PCR using *TmToll-7*-specific primers. As shown in Figures 4A,B, challenging larvae with *E. coli* or *S. aureus* resulted in similar time-course changes in *TmToll-7* mRNA levels, which became significantly upregulated at 6 h post-infection, and then decreased, but remained higher than control levels at 9 and 12 h post-infection. However, in the case of *C. albicans* infection, *TmToll-7* mRNA levels reached a maximum at 9 h post-infection, and then declined to control levels at 12 h post-infection (Figure 4C).

### *T. molitor* Larval Mortality Assay

**Figure 5:**
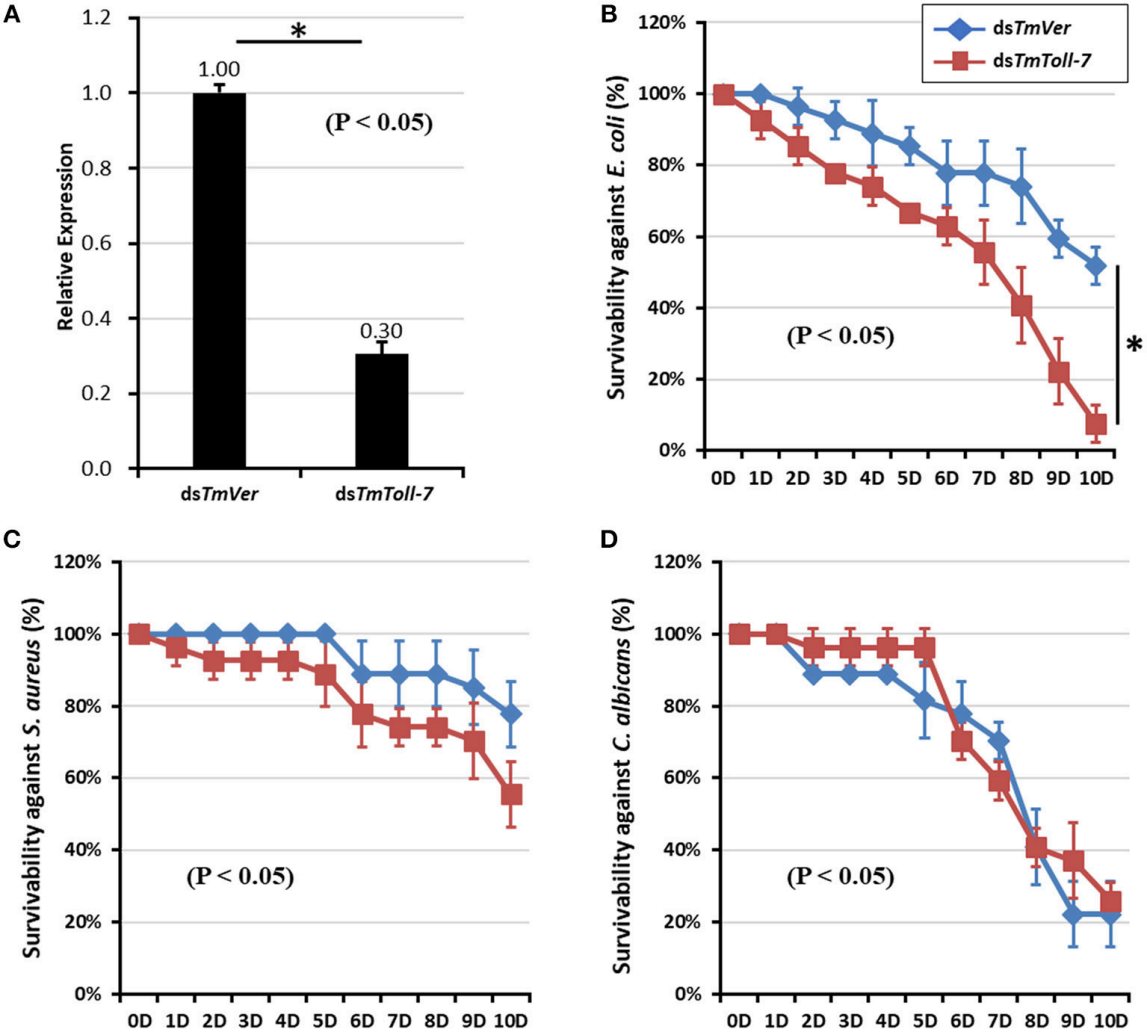
Effect of *TmToll-7* gene silencing on survivability of *T. molitor* larvae. **(A)** Down-regulated *TmToll-7* transcripts by RNAi. dsRNA for *TmToll-7* (2 μg per insect) was injected into 10~11^th^ instar larvae. To determine knockdown levels of *TmToll-7* transcripts, quantitative PCR was performed by using total RNA isolated from 10~11th instar larvae (n = 3). The transcripts levels of *TmToll-7* was reduced at 7-days after *TmToll-7* RNAi. “^*^” indicates a significant (*p* < 0.05) difference between control treatments. Survival curves of *T. molitor* larvae infected with *E.coli* **(B)**, *S. aureus* **(C)**, *C. albicans* **(D)** following down-regulation of *TmToll-7* mRNA transcripts. At the end of the experimental period (10 days post-infection), the survival rates of larvae were 7% at 10 h post-*E. coli* infection and 52% in *TmToll-7*-depleted larvae compared to the ds*TmVer* control group.

Since we found *TmToll-7* expression to be induced after infection by *E. coli, S. aureus*, or *C. albicans*, we next wanted to determine if TmToll-7 plays a role in resistance to bacteria and fungi by monitoring the survival rates of infected *T. molitor* larvae after treatment with either control dsRNA (*TmVer*) or *TmToll-7* dsRNA. Figure 5A shows that 7 days after injection, *TmToll-7* mRNA levels decreased by 70% in larvae treated with *TmToll-7* dsRNA compared to those treated with control dsRNA. After confirming efficient knockdown, we then challenged ds*TmToll-7*-treated and control larvae by injecting them with 1 μl of bacterial (*E. coli* or *S. aureus*, 1 × 10^6^/μl) or fungal suspension (*C. albicans*, 5 × 10^4^/μl) and followed their survival for 10 days. We found that compared to dsTmVer (control) larvae, ds*TmToll-7* larvae were significantly more susceptible to *E. coli* infection (93 vs. 49% mortality) (Figure 5B), whereas their survival rates after infection with *S. aureus* or *C. albicans* were not significantly different from the controls (Figures 5C,D).

### Effects of *TmToll-7* RNAi on Expression of AMP and Other Downstream Signaling Genes in Response to Microorganism Infection

**Figure 6:**
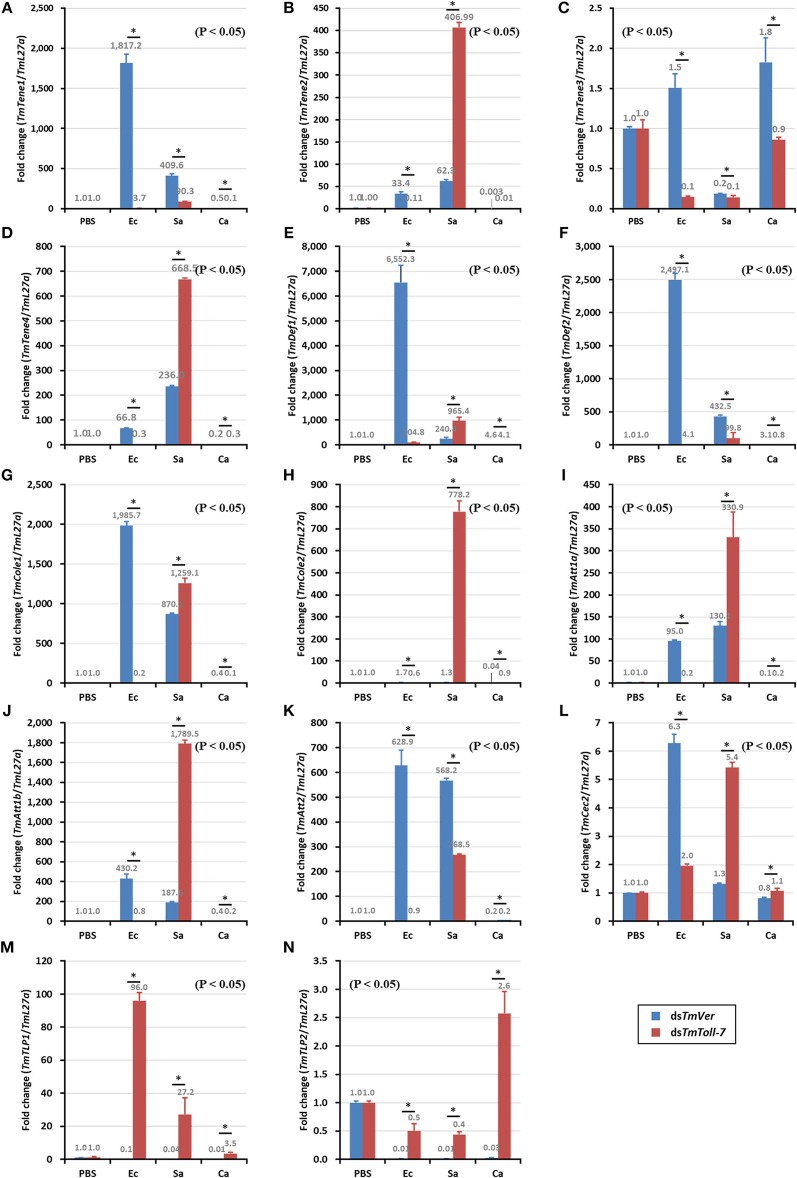
Induction patterns of 14 AMP genes in response to pathogenic microbial injection in *TmToll-7* silenced *T. molitor* larvae. *E. coli, C. albicans*, and *S. aureus* were injected into ds*TmToll-7*-teated *T. molitor* larvae and whole body samples were collected at 24 h post injection. Expression of AMP genes, including *TmTene-1* (**A**, *TmTenecin-1*); *TmTene-2* (**B**, *TmTenecin-2*); *TmTene-3* (**C**, *TmTenecin-3*); *TmTene-4* (**D**, *TmTenecin-4*); *TmDef-1* (**E**, *TmDefensin-1*); *TmDef-2* (**F**, *TmDefensin-2*); *TmCole-1* (**G**, *TmColeoptericin-1*); *TmCole-2* (**H**, *TmColeoptericin-2*); *TmAtt-1a* (**I**, *TmAttacin-1a*); *TmAtt-1b* (**J**, *TmAttacin-1b*); *TmAtt-2* (**K**, *TmAttacin-2*); *TmCec-2* (**L**, *TmCecropin-2*); *TmTLP-1* (**M**, *TmThaumatin-like protein-1*); and *TmTLP-2* (**N**, *TmThaumatin-like protein-2*) were investigated by using qPCR. *TmVer* dsRNA was used as a knock down control and *T. molitor ribosomal protein* (*TmL27a*) was used as an internal control. PBS, PBS-injected control; Ec, *E. coli*-injected; Sa, *S. aureus* injected; Ca, *C. albicans* injected.

Since we found that *TmToll-7* knockdown rendered *T. molitor* larvae significantly more susceptible to *E. coli* infection, but had no significant effect on survival rates after *S. aureus or C. albicans* infection, our data suggested that TmToll-7 is important for defense against Gram-negative bacteria. Thus, we next wanted to determine if TmToll-7 mediates this protective effect through AMP production. To do so, we knocked down *TmToll-7* once again and measured the expression levels of 14 different AMP genes following challenge with *E. coli, S. aureus*, or *C. albicans*. What we hoped to find in this experiment were AMPs that were significantly induced upon infection by *E. coli*, but reversed by *TmToll-7* knockdown. If so, this would suggest that TmToll-7, at least in part, mediates the activation of these AMPs in response to *E. coli* infection. Our data shows that among the 14 AMPs tested, *TmTenecin-1, TmDefensin-1, TmDefensin-2, TmColeroptericin-1*, and *TmAttacin-2* gene expression was induced in response to *E. coli* infection, but pretreatment with *TmToll-7* dsRNA suppressed their upregulation (Figures 6A,E–G,K). Interestingly, and in contrast to these effects, *TmToll-7* knockdown increased the mRNA levels of *TmTenecin-2, TmTenecin-4, TmColeoptericin-2, TmAttacin-1a*, and *TmAttacin-1b* in *S. aureus*-challenged larvae (Figures 6B,D,H–J). Finally, none of the 14 AMPs showed significant responses to *C. albicans* infection, regardless of whether or not *TmToll-7* was knocked down prior to infection.

To further examine the role of TmToll-7 in regulating the immune response against *E. coli*, we investigated how *TmToll-7* RNAi might affect the expression of six Toll pathway-related genes (*TmMyD88, TmTRAF, TmCactin, TmCactus, TmDorsal-1*, and *TmDorsal-2*) and one Imd-related gene (*TmRelish*) using specific primers listed in Supplementary Table 1. Our results showed that, except for *TmTRAF* and *TmDorsal-2*, the expression of the other four Toll pathway-related genes were significantly reduced by *TmToll-7* RNAi (*p* < 0.05), while the level of *TmRelish* was not strongly affected (Supplementary Figure 2; Supplementary Table 1). This suggests that TmToll-7 can positively regulate downstream genes of the Toll signaling pathway, and moreover, that TmToll-7 functions through the Toll signaling pathway to regulate AMP expression.

### Antimicrobial Activity Was Lost by ds*TmToll-7* Treatment

**Figure 7:**
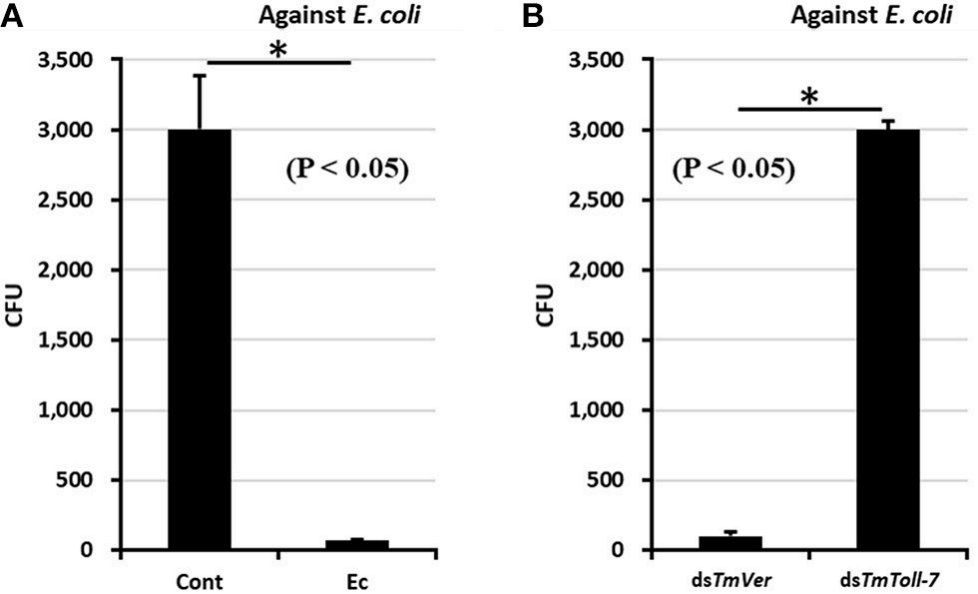
Antimicrobial activity assay against *E. coli* with *TmToll-7*-silenced *T. molitor* hemolymph by CFU method. **(A)** Antimicrobial activity was induced by *E. coli* (10^6^ cells/μl) injection. PBS-injected *T. molitor* hemolymph was used as a negative control. *E. coli*-injected *T. molitor* hemolymph had high antimicrobial activity compared with control hemolymph. Cont; assayed with PBS-injected *T. molitor* hemolymph, Ec, assayed with *E. coli*-injected *T. molitor* hemolymph **(B)** *E. coli* (10^6^ cells/μl) was injected into *TmToll7*-depleted *T. molitor* larvae. ds*TmVer*-treated *T. molitor* larvae was used as a negative control. The result indicates that the antimicrobial activity was dramatically decreased by treatment of ds*TmToll-7* compared with the ds*TmVer*-treated group. ds*TmVer*, assayed with *E. coli*-injected *T. molitor* hemolymph after treatment with ds*TmVer*; ds*TmToll-7*, assayed with *E. coli*-injected *T. molitor* hemolymph after treatment with ds*TmToll-7*.

Since knockdown of *TmToll-7* by RNAi clearly suppressed *E. coli*-induced AMP expression, we wanted to determine whether this suppression would result in greater bacterial proliferation in the insect's hemolymph. To test this, we performed colony-forming unit assays. First, we pretreated larvae with *TmToll-7* dsRNA or *TmVer* dsRNA (negative control) and infected them with *E. coli* to induce their immune system. After 24 h post-infection, cell-free hemolymph samples were prepared from these larvae and re-incubated with fresh *E. coli* for 2 h before being diluted and plated on LB agar for CFU counting. We found that pre-exposure to *E. coli* inhibited bacterial growth in the hemolymph of uninjected and TmVer dsRNA-injected control larvae, but not in the hemolymph of *TmToll-7* dsRNA-injected larvae since it gave high *E. coli* counts, equal to those obtained from control larvae that had been pre-exposed to PBS instead (Figures 7A,B). By contrast, when we screened the hemolymph for antimicrobial activity against *S. aureus* (Gram-positive) and *C. albicans* (fungi), we found that RNAi of *TmToll-7* did not significantly change the CFU numbers for both pathogens, i.e., the CFUs were low and comparable to the *TmVermilion* dsRNA-infected controls (~300 and 35 CFU, respectively) (Supplementary Figure 1). These results indicate that *TmToll-7* knockdown suppresses anti-Gram negative activity and the ability to clear *E. coli* from the hemolymph.

## Discussion

Using BLASTP with TmToll7 sequence as a query, we found more than 200 additional homologs in other insect species, with identities ranging from 48 to 93% (data not shown), suggesting that Toll-7 is widely conserved and essential in insects. This includes Toll-7, 18-wheeler, Toll-6 from *Drosophila*, which so far are the only 3 homologs (out of the 200) for which functional data are available (6–8, 10, 16). Overall, TmToll-7 shares 53, 51, and 43% sequence identity, respectively, with these three proteins, and like them, it has an N-terminal leucine-rich repeat (LRR) domain that is longer (~700 residues with 28 repeats) compared to other *Drosophila* Tolls (17 repeats in Toll-1, 2 in Toll-3, 17 in Toll-4, and 12 in Toll-5).

Although it is known, at least for Toll-1, that its LRR domain (specifically the first 13 LRRs) is required for formation of an active signaling complex (i.e., two Spz molecules bind to the LRR domains of two Toll receptor molecules, inducing a conformational change that enables dimer formation) (41, 42), the roles of the remaining Toll receptors (18-wheeler/Toll-2 to Toll-9, or any other insect TLR for that matter), are currently unclear. For example, it is unclear if and how they function in immunity or what ligands are required for their activation. However, it was recently shown in one study that Toll-7 and Toll-6 bind to and function together with Spätzle paralogs DNT-1/Spz-2 and DNT-2/Spz-5 in the *Drosophila* CNS to regulate motor-axon targeting and neuronal survival (10). Interestingly, the authors of this study also showed that in S2 cells, when either Toll-6 or 7 were stimulated with DNT-2 ligand, this activated the expression of a *drosomycin* reporter gene, which led them to suggest that Toll-6 and 7 could induce NF-kb signaling, possibly by using downstream signaling components, such as dMyD88, which are required by Toll-1 during embryogenesis and immunity (10). However, they ruled out the possibility that both receptors might have functional roles in immunity similar to Toll-1, since studies by Tauszig et al. and McIlroy et al. showed that Toll-6 and 7 do not activate the *drosomycin* reporter gene after immune challenge (10, 16).

It should be noted, however, that in the study by Tauszig et al. they examined the induction of the *drosomycin* reporter using chimeric constructs of Toll-2 to -8, in which the transmembrane and cytoplasmic domains of each receptor (devoid of their extracellular LRR domain) were fused to a truncated Toll-1 ectodomain. This was done for the purpose of determining if the cytoplasmic TIR domains of these receptors might be capable of activating antimicrobial peptide promoters and exhibit the same signaling specificity as Toll-1 (16). While their study showed that none of the chimeric proteins significantly induced expression of antimicrobial peptide genes, except for Toll-5, which activated drosomycin expression (16), we cannot help but wonder if one of the reasons why there is a discrepancy between Tauszig et al.'s (16) results and McIlroy et al.'s (10) is because Toll-6 and Toll-7 rely on their own extracellular LRR domain for ligand binding and signaling (the corresponding domain from Toll-1 cannot be substituted). If indeed they are required, this might explain why McIlroy et al. saw an induction in *drosomycin* promoter activity when they added purified DNT-2/Spz-5 protein to Toll-6 and Toll-7 expressing S2 cells.

We must add that, while writing this section, a preprint paper by Yu et al. showed that Toll-7, in its full-length (i.e., non-chimeric) form, is also capable of activating the drosomycin promoter when co-expressed with Spz-1 or Spz-2 (43). Importantly, they used co-immunoprecipitation assays to show that this activation may be mediated, in part, by binding of Toll-7, through its extracellular domain, to Spz-1, Spz-2, or Spz-5 (43). Given this evidence, and the fact that TmToll-7 shares 65% amino acid identity in its extracellular domain with Toll-7, there is reason to suggest that TmToll-7 binds to similar ligands as Toll-7.

Incidentally, a previous RNA-sequencing study conducted with bacterial-induced transcripts from *T. molitor* adults identified 9,570 putative orthologs of previously annotated *T. castaneum* genes, 213 of which were classified as immune-related (44). Interestingly, among these, an ortholog of Toll-7, one of only two Toll genes, the other being an ortholog of Toll-3, was identified along with seven Spätzle-like protein encoding genes (Spz 1–7). In addition to *spz*-3, 4, 6, and 7, our group has also recently identified five more spätzle-like genes: four are homologous to *Tribolium spz-1b, spz-like, spz-7a*, and *spz-7b*, the latter two of which encode 141- and 185-amino acid isoforms of Spz-7, and one is homologous to *Drosophila* Spz-5. We are now in the process of determining which of these ligands are required for AMP expression through knockdown studies. Of the nine ligands listed above, we have so far looked at three (*TmSpz-7, -7a*, and *-7b*), and our preliminary studies indicate that RNAi of *TmSpz-7* and *-7b* reduces the induction of several AMPs, including the five aforementioned AMPs (Cho et al., unpublished results), which we have demonstrated here to be suppressed by *TmToll-7* RNAi after *E. coli* challenge. It will be interesting to see, in future studies, whether either of these ligands binds directly to *TmToll-7* (or other receptors) and, if they do, whether their interaction is relevant to immunity and/or development in *T. molitor*.

Although generally recognized as being responsive to Gram-positive bacteria and fungi, the Toll pathway in *Drosophila* has recently been demonstrated by Duneau et al. (45) and Yu et al. (43) to be sexually dimorphic in response to Gram-negative bacteria infection (43, 45). In the Duneau et al. study, they found that the induction of *Drosomycin* was much stronger in wild-type males than in females in response to Gram-negative *Providencia rettgeri* infection; however, this dimorphism was lost in *spätzle* and *persephone* mutants, but not in *modSP* mutants. Based on these results, Duneau et al. hypothesized that, in the case of *P. rettgeri* infection, Toll pathway activation specifically occurring via the Persephone branch leads to higher expression of AMP genes in males. In the study by Yu et al. upon observing that Toll-1 is expressed in both adult males and females while Toll-7 is expressed in males only, they looked at the susceptibility of Toll-7 mutant males to Gram-negative *Pseudomonas aeruginosa* infection and found that they were significantly more susceptible than Toll-1 mutant males. Together, these differential effects suggest that Toll-7 plays a major role in defense against *P. aeruginosa* infection in adult males only (43). However, it should be noted in our study, although we found that *TmToll-7* was induced in *Tenebrio* adults (males and females combined) in response to Gram-negative *E. coli* infection, it was to a lesser extent than that observed in infected larvae. Thus, for the remainder of our studies, we focused on the effects of *TmToll-7* silencing in larvae, which rendered larvae more susceptible to *E. coli* infection and suppressed certain AMP genes induced by *E. coli* challenge (including *TmDefensin-1, TmDefensin-2, TmColeoptericin-1*, and *TmAttacin-2*, all four of which belong to AMP families characterized as having antibacterial activity against Gram-negative bacteria) (46–48). In addition to the above studies in *Drosophila, in vitro* reconstitution experiments showing that *E. coli* DAP-type peptidoglycans can induce cleavage of pro-ModSP and pro-Spätzle after incubation with purified *Tenebrio* PGRP-SA and GNBP1 (signaling components upstream of Toll) provide further evidence that *E. coli* is able to activate Toll signaling in *T. molitor* larvae, in this case, we suggest can occur via TmToll-7 (29). Moreover, these *in vitro* findings suggest that unlike in *Drosophila*, the *Tenebrio* Toll pathway can be activated by recognition of Gram-negative bacteria through cleavage of ModSP, although at this point we cannot rule out the possibility that Toll activation may also occur via the Persephone branch.

One general question that arises from this study is what makes TmToll-7 different from *Drosophila* Toll-1 and other insect Tolls that mediate immune responses to Gram-positive bacteria and fungi? To try and answer this question, we have further compared the TIR sequence of TmToll-7 with those of *Drosophila* 18W and Toll-7, given that they are closely related phylogenetically. Protein sequence alignments show that the TmToll-7 TIR domain shares 53% identity and 70% similarity with that of Toll-7 and 48% identity and 68% similarity with that of 18W. These similarities were higher than when compared with the TIR domain of Toll-1 (35% identity and 55% similarity), suggesting that TmToll-7 may activate similar intracellular signaling components as Toll-7 and 18W; but given that Toll-1 also shares a certain level of identity with TmToll-7, we cannot rule out the possibility that they too may share some conserved signaling components.

**Figure 8:**
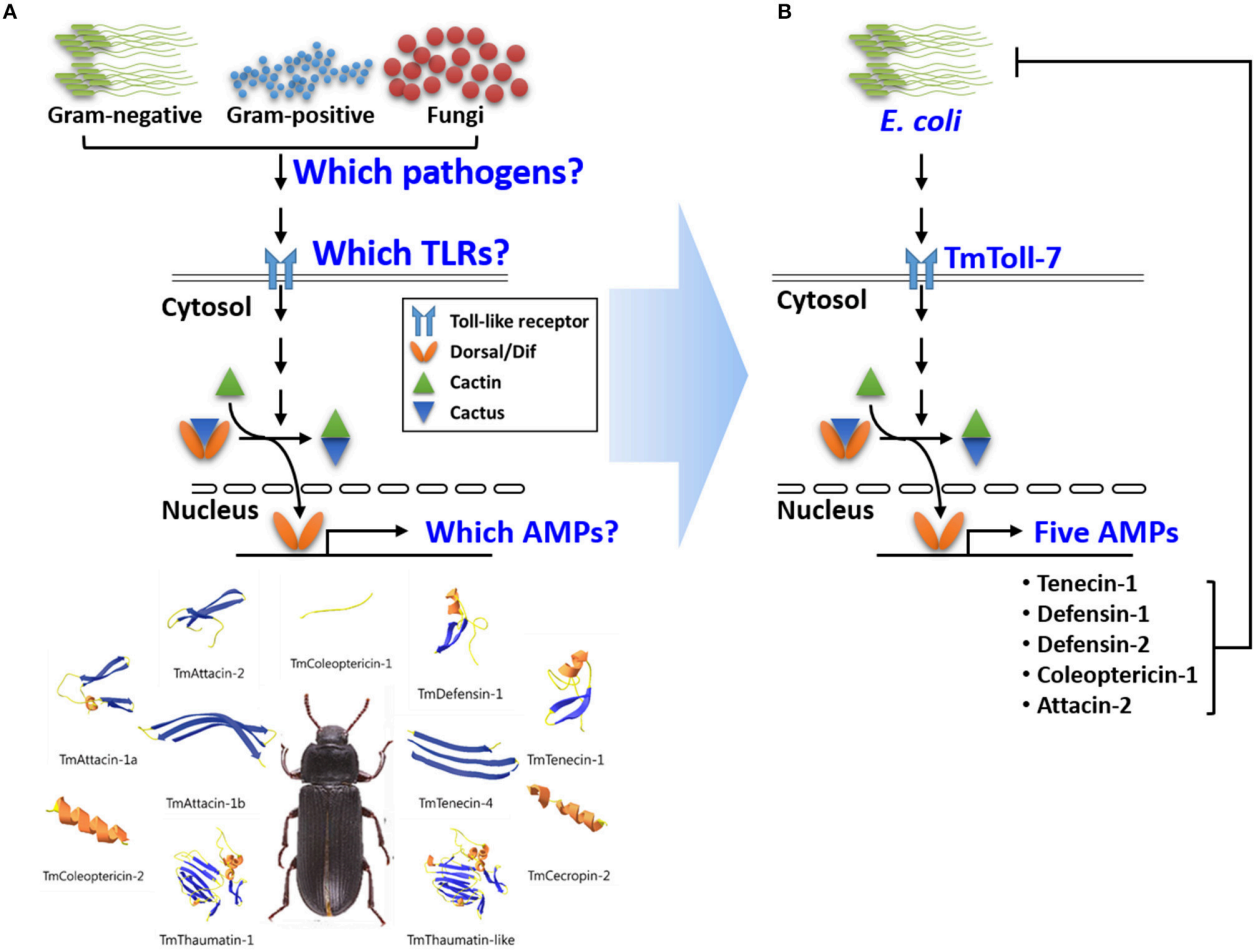
Graphical illustration of *TmToll-7* on Toll-signaling pathway in *T. molitor*. **(A)** The questions and **(B)** answers for our study. Our results suggest that the Gram-negative bacterium, *E. coli*, stimulates TmToll7, resulting in the induction of five AMP genes that kill *E. coli*.

While so far, none of the intracellular proteins that interact with and/or function downstream of Toll-7 and 18W have been identified, it is known, at least for 18W, that it is required for the nuclear import of the NF-kB transcription factor, Dif, and for the induction of the antibacterial peptide gene *attacin* (49). Moreover, studies have shown that the induction of *attacin* involves the Imd transcription factor Relish, in addition to Dif (i.e., Dif and Relish form a heterodimer that binds to the promoter of this antibacterial gene in order to regulate its expression) (50). Given that orthologs for most of the intracellular components of the Toll-1 and Imd pathways have been identified in *T. molitor*, including two Dif orthologs and a Relish ortholog (44), and based on our data above showing that *TmToll-7* RNAi inhibits *Tmattacin-2* expression, we hypothesize that similar to 18W, TmToll-7 in *T. molitor* plays a role in mediating nuclear translocation of Dif and that it also uses a Dif-Relish heterodimer for activating AMP gene expression. However, one major difference between these receptors may be that unlike 18W, TmToll-7 can be activated by Gram-negative bacteria (Figure 8) given that it has been demonstrated through *in vitro* experiments that DAP-type peptidoglycan of Gram-negative bacteria binds to the *Tenebrio* recognition protein PGRP-SA and induces proteolytic activation of Spätzle (29). In addition to *Tmattacin-2*, we also found that *Tmdefensin-1* and *Tmdefensin-2* were among the five AMPs that resulted in suppressed expression after *E. coli* challenge and *TmToll-7* RNAi. Earlier studies in *Drosophila* S2 cells have shown that Dif by itself is sufficient to activate *defensin* expression (51), and when in combination with Relish upregulates *defensin* activity (50), but whether this also occurs *in vivo* in *T. molitor* remains to be determined. Now that several NF-kB protein sequences (Dif1, Dif2, Rel1, and Rel2) from *T. molitor* have been identified, we can begin to perform further studies to determine which combinations of these Rel proteins are formed and which AMP promoters they directly bind to and regulate.

## Author Contributions

YH and YJ: conceived and designed the experiments; SP, KP, HK, CK, YB, and BK: performed the experiments; YJ and SP: analyzed the data; YH and YL: contributed reagents, materials, analysis tools; YH, YJ, SP, and YK: wrote the manuscript; SJ, IB, and YL: revised the manuscript.

### Conflict of Interest Statement

The authors declare that the research was conducted in the absence of any commercial or financial relationships that could be construed as a potential conflict of interest.
