# Supplementary material for: TmToll-7 Plays a Crucial Role in Innate Immune Responses Against Gram-Negative Bacteria by Regulating 5 AMP Genes in Tenebrio molitor
Source: Front Immunol. 2019 Mar 12;10:310. doi: 10.3389/fimmu.2019.00310 (PMC6424196; doi:10.3389/fimmu.2019.00310)
Supplement: Supplementary file 1 [file Data_Sheet_1.docx]

For Supplementary Material and Methods:

## 1. *TmToll-7* RNAi has critical role in antimicrobial activity against *E. coli*

The antimicrobial activity against *S. aureus* or *C. albicans* was assayed by CFU method as described in the Materials and Methods. *S. aureus* (10^6^ cells) or *C. albicans* (5 x 10^4^ cells) were injected into *TmToll-7* dsRNA-treated *T. molitor* larvae and hemolymph was used for this assay

## 2. Effects of *TmToll-7* RNAi on expression of downstream signaling genes in response to microorganism infection

In addition, the effects of TmToll-7 knockdown on the expression of Toll (*TmMyD88*, *TmTRAF*, *TmCactin*, *TmCactus*, *TmDorsal-1*, and *TmDorsal-2*) and Imd (*TmRelish*) pathway-related genes were examined by qRT-PCR as described in the Materials and Methods. It should be noted that since our AMP expression data showed that *TmToll-7* RNAi significantly reduced the transcription of five AMPs after *E. coli* challenge (but not after *S. aureus* or C. *albicans* challenge) (Fig. 6), in these sets of experiments, we focused on challenging TmToll-7 RNAi larvae with *E. coli* only and performing qRT-PCR on mRNA from these larvae. A list of specific primers used for qRT-PCR in this study can be found in Supplementary Table 1.

**Supplementary Table 1**. Primers used in the present study

| Name | Primer sequences |
| --- | --- |
| TmMyD88_qPCR_Fw  TmMyD88_qPCR_Rev | 5’-AGTGCTCAGACAGAAGACCA-3’  5’-TACGGTCCATCTCGTCCAAA-3’ |
| TmTRAF_qPCR_Fw  TmTRAF_qPCR_Rev | 5’-TCGCAGCAGAAAACCAAGTG-3’  5’-AGCGCAACTTCTCGTTTTCG-3’ |
| TmCactin_qPCR_Fw  TmCactin_qPCR_Rev | 5’-AAGCGGCGCAATTTGAAGAG-3’  5’-TCCGCGCTTATGTATTTCGC-3’ |
| TmCactus_qPCR_Fw  TmCactus_qPCR_Rev | 5’-ACCGTTCAACAGTGAAGACG-3’  5’-ACAACGTGCTCGGATTTTCG-3’ |
| TmDorsal1_qPCR_Fw  TmDorsal1_qPCR_Rv | 5’-AGCGTTGAGGTTTCGGTATG-3’  5’-TCTTTGGTGACGCAAGACAC-3’ |
| TmDorsal2_qPCR_Fw  TmDorsal2_qPCR_Rev | 5’-ACACCCCCGAAATCACAAAC-3’  5’-TTTCAGAGCGCCAGGTTTTG-3’ |
| TmRelish_qPCR-Fw  TmRelish_qPCR-Rev | 5’-AGCGTCAAGTTGGAGCAGAT-3’  5’-GTCCGGACCTCATCAAGTGT-3’ |


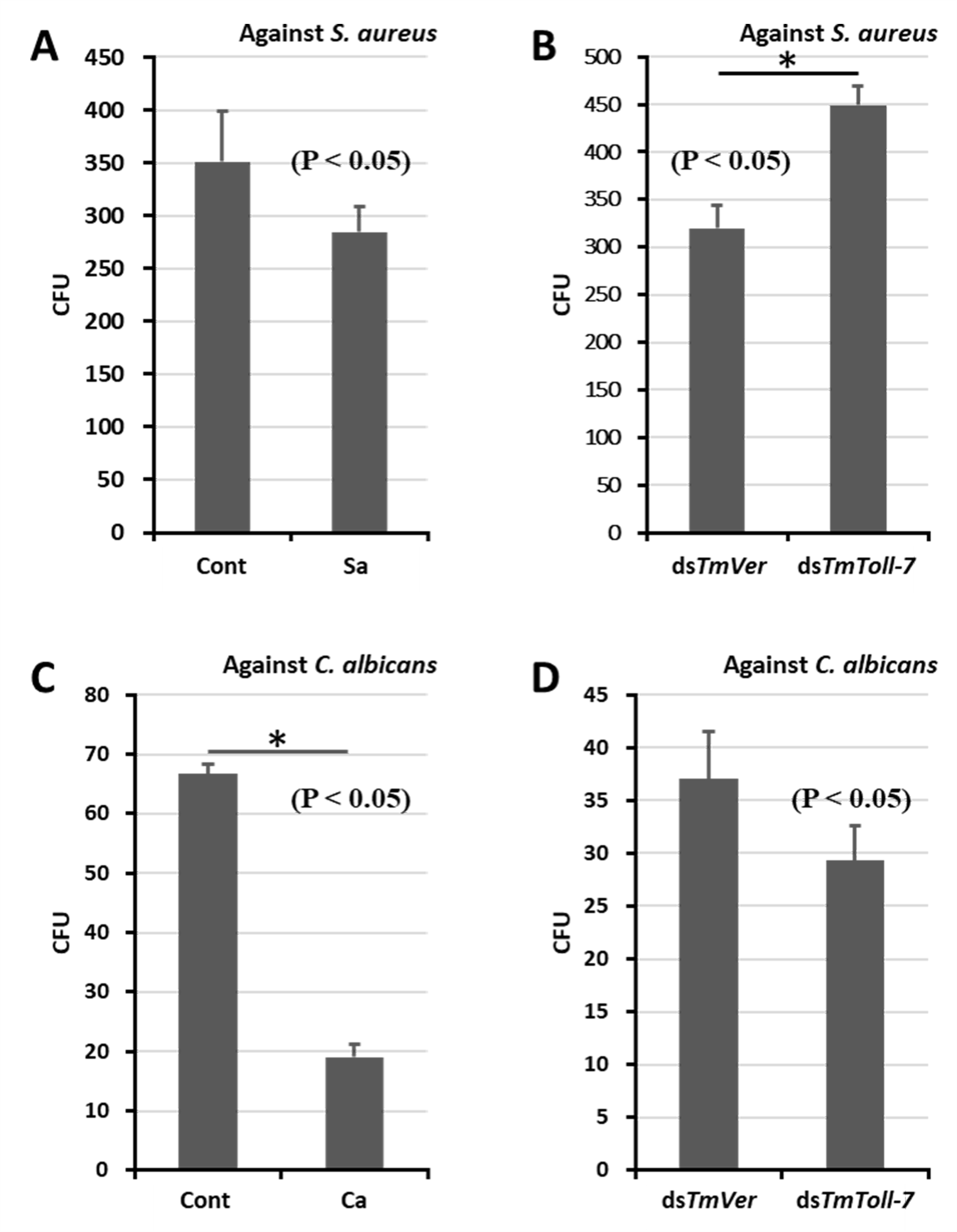


**Supplementary Figure 1.** Antimicrobial activity assay against *S. aureus* or *C. albicans* with *TmToll-7*-silenced *T. molitor* hemolymph by CFU method. (A, C) Antimicrobial activity was induced by *S. aureus* (A, 10^6^ cells/µl) or *C. albicans* (C, 5 × 10^4^ cells/µl) injection. PBS injected *T. molitor* hemolymph was used as a negative control. *S. aureus* or *C. albicnas* injected *T. molitor* hemolymph had increased antimicrobial activity compared with control hemolymph. Cont; Assayed with PBS injected *T. molitor* hemolymph, Sa; Assayed with *S. aureus* injected *T. molitor* hemolymph, Ca; Assayed with *C. albicans* injected *T. molitor* hemolymph (B, D) *S. aureus* (10^6^ cells/µl) or or *C. albicans* (D, 5 × 10^4^ cells/µl) was injected into *TmToll7*-depleted *T. molitor* larvae. ds*TmVer*–treated *T. molitor* larvae was used as a negative control. The result indicates that the antimicrobial activity against *S. aureus* was significantly decreased by treatment of ds*TmToll-7* compared with the ds*TmVer*-treated group. Whereas, the antimicrobial activity against *C. albicans* was not recovered by treatment of ds*TmToll-7* compared with the ds*TmVer*-treated group. ds*TmVer*; Assayed with *S. aureus* or *C. albicans* injected *T. molitor* hemolymph after treatment with ds*TmVer*, ds*TmToll-7*; Assayed with *S. aureus* or *C. albicans* injected *T. molitor* hemolymph after treatment with ds*TmToll-7*.


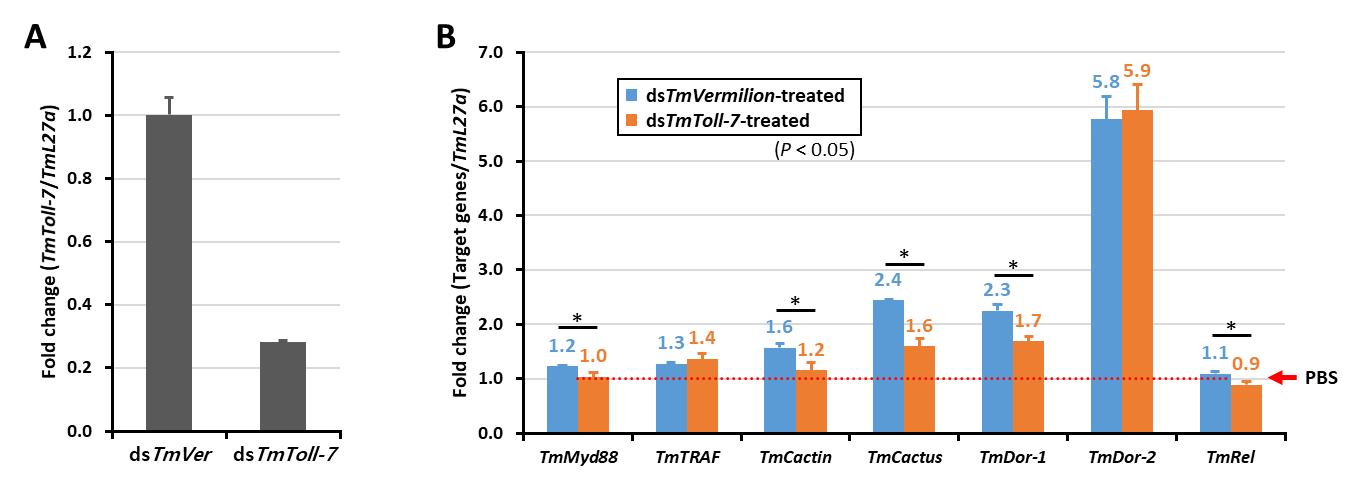


**Supplementary Figure 2.** Effects of TmToll-7 RNAi on expression of Toll and Imd pathway-related genes in response to E. coli infection. (A) Decreased expression of TmToll-7 (about 72%) was detected by TmToll-7 RNAi. (B) E. coli was injected into dsTmToll-7-teated T. molitor larvae and whole body samples were collected at 24 h post injection. Expression of Toll (TmMyD88, TmTRAF, TmCactin, TmCactus, TmDorsal-1, and TmDorsal-2) and Imd (TmRelish) pathway-related genes were investigated by using qRT-PCR. TmVer dsRNA and PBS were used as a knock down and injection controls, respectively, and T. molitor ribosomal protein (TmL27a) was used as an internal control.
